# Supplementary material for: How value perspectives influence decision-making in the South African private healthcare sector: A cross-sectional comparative study
Source: PLoS One. 2025 Feb 4;20(2):e0316547. doi: 10.1371/journal.pone.0316547 (PMC11793816; doi:10.1371/journal.pone.0316547)
Supplement: S1 File — (DOCX) [file pone.0316547.s001.docx]

**Supplementary Material**

**Appendix 1 Value Perspectives Survey (VPS)**

This document outlines the Value Perspectives Survey (VPS) that was administered to the participants using the Qualtrics platform. Notes concerning the design and flow of the VPS are indicated as ‘**Note:**’ below relevant questions (these notes were not viewed by the participants). Questions marked with an asterisk (*) were compulsory.

**Doctor or Patient Perspective**

**Are you completing this survey from the perspective of a doctor or a patient?***

| Doctor | 1 |
| --- | --- |
| Patient | 2 |

**Note:** Based on the participant’s response, he or she was directed to the Patient Survey or Doctor Survey section.

**Patient Survey**

**Basic demographic information section**

**Do you use private healthcare in South Africa?***

| I only use public healthcare and never private healthcare | 1 |
| --- | --- |
| I sometimes use private healthcare and sometimes use public healthcare | 2 |
| I only use private healthcare and never public healthcare | 3 |

**Note:** If a participant selected option 1 (“I only use public healthcare and never private healthcare”), he or she was directed to the end of the survey. When the data was cleaned prior to statistical analysis, the participants who had selected option 1 were removed from the dataset.

**Gender**

| Male | 1 |
| --- | --- |
| Female | 2 |
| Prefer not to say | 3 |

**Age in years**

|  |
| --- |

**What is your highest level of education?**

| Less than Grade 12 | 1 |
| --- | --- |
| Grade 12 | 2 |
| Diploma | 3 |
| University degree (bachelor degree) | 4 |
| Post graduate honours degree | 5 |
| Post graduate master’s degree | 6 |
| Post graduate doctoral degree | 7 |
| Other | 8 |

**What is your total gross monthly household income?**

| < R10 000 | 1 |
| --- | --- |
| R10 000 – R19 999 | 2 |
| R20 000 – R29 999 | 3 |
| R30 000 – R39 999 | 4 |
| R40 000 – R49 999 | 5 |
| R50 000 – R19 999 | 6 |
| R60 000 – R29 999 | 7 |
| More than R69 999 | 8 |
| Prefer not to say | 9 |

**Are you a member of a medical aid?***

| Yes | 1 |
| --- | --- |
| No | 2 |

**Note:** If participants answered ‘No’, the following two questions were skipped.

**How long have you been a member of a medical aid?***

| Longer than 10 years | 1 |
| --- | --- |
| 6 – 10 years | 2 |
| 1 – 5 years | 3 |
| Less than a year | 4 |

**How much does it cost for you to be on a medical aid per month? State amount in South African Rands (ZAR), rounded off to the nearest hundred.***

|  |
| --- |

**Are you financially responsible for payment for private medical care/medical aid you or your family receives?***

| I am the sole contributor towards all medical costs for myself/and or my family | 1 |
| --- | --- |
| I partly contribute towards medical costs for myself/and or my family | 2 |
| I do not contribute towards any medical costs for myself/and or my family | 3 |

**Value Perspectives Section**

**General**

Every clinical care event aims to create value at a certain cost. For this study, a clinical event refers to a hospital admission for a medical or surgical treatment. The next set of questions are aimed at exploring the differences between the relative importance of three factors (Clinical Outcome, Cost of Clinical Event, and Patient’s Experience) that make up the Value of Care delivered to patients:

- 'Clinical Outcome’ is the degree to which the clinical event achieved a clinical goal (objective measure);
- ‘Cost of the Clinical Event’ is the total price charged by the care providers, thus the price paid by the patient or medical aid or both;
- ‘Patient’s Experience’ is the degree to which the patient’s expectation was met (subjective measure).

From your perspective, please indicate how important each of these factors (Clinical Outcome, Cost of Clinical Event, and Patient’s Experience) are in ensuring that high value care is delivered to patients.

**Value of Care**

(where 0 = not important at all and 100 = extremely important)

From your perspective, please indicate how important each of these factors (Clinical Outcome, Cost of Clinical Event, and Patient’s Experience) are by allocating a total of 100% between the three value factors. For example, you can allocate 20% to Clinical Outcomes, 30% to Cost of Clinical Event and 50% to Patient’s Experience. The total score must equal 100%.*

| Clinical Outcome | 0 - 100 |
| --- | --- |
| Cost of the Clinical Event | 0 - 100 |
| Patient’s Experience | 0 - 100 |

**Note:** A sliding scale functionality was used for all general, surgical and medical scenarios to ensure ease of allocation of points (see Figure 2).

**
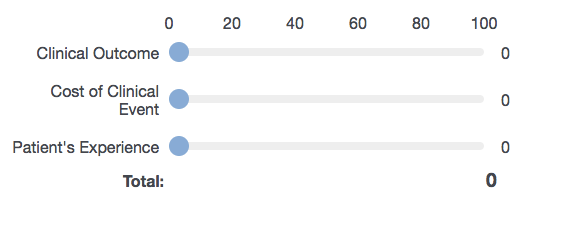
**

Figure 2. Screenshot (webpage view as accessed via desktop or laptop computer or tablet) of sliding scale functionality used for general, clinical and medical scenario value perspective sections.

**Clinical Scenarios**

In the next section, you will be asked to compare all three of these value factors in a series of six clinical scenarios.

From your perspective, please indicate how important each of these factors (Clinical Outcome, Cost of Clinical Event and Patient’s Experience) are by allocating a total of 100% between the three value factors. For example, you can allocate 20% to Clinical Outcomes, 30% to Cost of Clinical Event and 50% to Patient’s Experience. The total score must equal 100% for every scenario.

**Scenario 1:** You require admission to a day ward for surgery to remove a skin tag.*

**Scenario 2:** You require admission to a general ward for surgery to repair a hernia in your groin.*

**Scenario 3:** You require admission to an intensive care unit for abdominal surgery for the treatment of liver cancer that has spread to other organs in your body.*

**Scenario 4:** You require admission to a day ward for control of your blood sugar levels that are too high.*

**Scenario 5:** You require admission to a general ward to receive oxygen therapy for pneumonia.*

**Scenario 6:** You require admission to an intensive care unit for a heart attack with severe shock as complication.*

Thank you for your time!

**Note:** For every clinical scenario question, the three factors (Clinical Outcome, Cost of Clinical Event, and Patient’s Experience) appeared in a randomised sequence, thereby assuring that the same factor did not always appear last in the sequence of options provided to the participant.

Following completion of the six scenario-based questions the participant was thanked for his or her time and exited the survey.

**Doctor Survey**

**Basic demographic information section**

**Do you practice medicine in South Africa or another country?***

| I practice only in South Africa | 1 |
| --- | --- |
| I practice in South Africa and another country | 2 |
| I do not practice in South Africa | 3 |

**Note:** Should a respondent select option 3 (“I do not practice in South Africa”), he or she was directed to the end of the survey. When the data was cleaned prior to statistical analysis, the participants who had selected option 3 were removed from the dataset.

**Gender**

| Male | 1 |
| --- | --- |
| Female | 2 |
| Prefer not to say | 3 |

**Age in years**

|  |
| --- |

**Are you a General Practitioner or a Specialist?***

| General Practitioner | 1 |
| --- | --- |
| Specialist | 2 |

**Do you practice clinical medicine or do you work in a managerial or administrative capacity as a doctor?***

| I practice clinical medicine | 1 |
| --- | --- |
| I work a manager or in administrative capacity as a doctor | 2 |

**If you practice clinical medicine, do you practice a medical, surgical (including anaesthetics) or diagnostic (including radiology and pathology) discipline?***

| Medical | 1 |
| --- | --- |
| Surgical | 2 |
| Diagnostic | 3 |
| Not applicable | 4 |

**Do you work in the private or public healthcare sector or a combination of the two?***

| Private healthcare sector | 1 |
| --- | --- |
| Public healthcare sector | 2 |
| Combination of the two | 3 |

**How many years have you been practicing medicine or working in healthcare in South Africa (excluding internship and community service)?***

|  |
| --- |

**Of the years that you have been practicing medicine or working in healthcare (excluding internship and community service), how many have been in the private sector?***

|  |
| --- |

**Value Perspectives Section**

**General**

Every clinical care event aims to create value at a certain cost. For this study, a clinical event refers to a hospital admission for a medical or surgical treatment. The next set of questions are aimed at exploring the differences between the relative importance of three factors (Clinical Outcome, Cost of Clinical Event, and Patient’s Experience) that make up the Value of Care delivered to patients:

- 'Clinical Outcome’ is the degree to which the clinical event achieved a clinical goal (objective measure);
- ‘Cost of the Clinical Event’ is the total price charged by the care providers, thus the price paid by the patient or medical aid or both;
- ‘Patient’s Experience’ is the degree to which the patient’s expectation was met (subjective measure).

From your perspective, please indicate how important each of these factors (Clinical Outcome, Cost and Patient’s Experience) are in ensuring that high value care is delivered to patients.

**Value of Care**

(where 0 = not important at all and 100 = extremely important)

From your perspective, please indicate how important each of these factors (Clinical Outcome, Cost of Clinical Event and Patient’s Experience) are by allocating a total of 100% between the three value factors. For example, you can allocate 20% to Clinical Outcomes, 30% to Cost and 50% to Patient’s Experience. The total score must equal 100%.*

| Clinical Outcome | 0 - 100 |
| --- | --- |
| Cost of the Clinical Event | 0 - 100 |
| Patient’s Experience | 0 - 100 |

**Note:** A sliding scale functionality was used for all general, surgical and medical scenarios to ensure ease of allocation of points (see Figure 2).

**Clinical Scenarios**

In the next section, you will be asked to compare all three of these value factors in a series of six clinical scenarios.

From your perspective, please indicate how important each of these factors (Clinical Outcome, Cost of Clinical Event and Patient’s Experience) are by allocating a total of 100% between the three value factors. For example, you can allocate 20% to Clinical Outcomes, 30% to Cost of Clinical Event and 50% to Patient’s Experience. The total score must equal 100% for every scenario.

**Scenario 1:** Your patient requires admission to a day ward for the surgical removal of a skin tag.*

**Scenario 2:** Your patient requires admission to a general ward for the surgical repair of an inguinal hernia.*

**Scenario 3:** Your patient requires admission to an intensive care unit for abdominal surgery for the treatment of metastatic hepatic carcinoma.*

**Scenario 4:** Your patient requires admission to a day ward for control of hyperglycaemia.*

**Scenario 5:** Your patient requires admission to a general ward for a community acquired pneumonia for oxygen therapy.*

**Scenario 6:** Your patient requires admission to an intensive care unit for a myocardial infarction with cardiogenic shock.*

Thank you for your time!

**Note:** For every clinical scenario question, the three factors (Clinical Outcome, Cost of Clinical Event, and Patient’s Experience) appeared in a randomised sequence, thereby assuring that the same factor did not always appear last in the sequence of options provided to the participant.

Following completion of the six scenario-based questions the participant was thanked for his or her time and exited the survey.

**Appendix 2.** Pearson correlations for value perspectives of consumers and providers across general, medical and surgical scenarios

|  | 1 | 2 | 3 | 4 | 5 | 6 | 7 | 8 | 9 |
| --- | --- | --- | --- | --- | --- | --- | --- | --- | --- |
| 1. General Clinical Outcome | - | -0.600** | -0.762** | 0.533** | -0.308** | -0.389** | 0.499** | -0.328** | -0.429** |
| 1. General Cost | **-0.674**** | - | -0.061 | -0.430** | 0.507** | 0.012 | -0.403** | 0.444** | 0.132* |
| 1. General Patient experience | **-0.692**** | **-0.066** | - | -0.321** | -0.029 | 0.484** | -0.300** | 0.048 | 0.435** |
| 1. Surgical Clinical Outcome | **0.570**** | **-0.441**** | **-0.364**** | - | -0.711** | -0.573** | 0.575** | -0.525** | -0.319** |
| 1. Surgical Cost | **-0.432**** | **0.589**** | **0.044** | **-0.688**** | - | -0.169** | -0.450** | 0.633** | -0.016 |
| 1. Surgical Patient experience | **-0.348**** | **0.013** | **0.456**** | **-0.682**** | **-0.062** | - | -0.281** | -0.003 | 0.466** |
| 1. Medical Clinical Outcome | **0.505**** | **-0.429**** | **-0.289**** | **0.726**** | **-0.551**** | **-0.443**** | - | -0.797** | -0.693** |
| 1. Medical Cost | **-0.373**** | **0.522**** | **0.027** | **-0.560**** | **0.695**** | **0.070** | **-0.724**** | - | 0.117* |
| 1. Medical Patient experience | **-0.344**** | **0.079** | **0.391**** | **-0.470**** | **0.076** | **0.570**** | **-0.697**** | **0.010** | - |
