# Supplementary material for: How value perspectives influence decision-making in the South African private healthcare sector: A cross-sectional comparative study
Source: PLoS One. 2025 Feb 4;20(2):e0316547. doi: 10.1371/journal.pone.0316547 (PMC11793816; doi:10.1371/journal.pone.0316547)
Supplement: Attachment — Submitted filename: Plos One___Peer Revewer 1.pdf [file pone.0316547.s002.pdf]

How value perspectives influence decision-making in the South African private healthcare sector: a cross-sectional comparative study

Peer Reviewer Comments

<u>MAJOR COMMENTS</u>

Abstract

1. The abstract should have an introductory sentence, followed by the objectives and gaps identified. This will make the content of the background under the Abstract section meaningful. Also, include the statistical figures for the results sub-section, especially the significant findings. The formula for the relationship stated under the 'Result' sub-section can be written as:

   VCI = (Outcome ÷ Cost) x Patient experience

   instead of expressing it as the

   Value Care Index (VCI): $VCI = \frac{outcome}{\text{cost}}\, x\ patient\ experience$

   Kindly rewrite the entire abstract and remove unambiguous information from the abstract. Let it be concise and meaningful.

Introduction

2. The introduction is very short and did not include much vital information on the subject matter. The introduction is the place to review other conclusions on the topic. The introduction section should introduce past findings to those who might not have that expertise. The introduction should have steps such as: introduce the topic. The first phase of the introduction is to tell the reader what the topic is and why it's interesting or important by describing the background; establishing the research problem; specifying the objective(s); and mapping out the paper. Also, remember that there are general phases that are associated with writing an introduction: stating the intent of the study, outlining the key characteristics of the study, describing important results, and giving a brief overview of the structure of the paper. Note that, the introduction of this study should follow the 'Funnel Shape' narrative method to scope and review existing studies across the worldwide and the study setting. This will illustrate the trends, pattens and prevalence rates of the outcome of interests of this study. Kindly revise.

3. Where are the gaps identified in this study? What has the previous studies have mentioned as regards the outcome(s) of this study? What is the current study saying on the current situation of your study outcome(s)? Always remember that a gap in a research study is an unanswered question or unresolved problem in a field, which reflects a lack of existing research in that space or it can exist when there is a concept or new idea that hasn't been studied at all, when the existing research is outdated or insufficient, or when there is a disagreement, contextual or methodological issue among the researchers. A gap in a research study indicates an opportunity for further investigation and contribution to the scientific knowledge. Kindly revise.

4. Where are the main objective of this study? Where are the specific objectives of this study? Kindly state them clearly.

5. The Keywords are so funny in nature…you have to follow the Journal format of presenting Keywords for your study. However, I may suggest the Keywords should be: Clinical outcome, patient experience, healthcare, South Africa, value perspectives, instead of you have below:

   Keywords - Value of care; clinical outcome; cost of clinical event; patient experience; value perspectives; private healthcare; South Africa

6. Kindly re-write the formula for the relationship stated throughout the entire manuscript as:

   VCI = (Outcome ÷ Cost) x Patient experience

   instead of expressing it as the

   Value Care Index (VCI): $VCI = \frac{\underline{outcome}}{\text{cost}}\ x\ patient\ experience$

   Kindly revise the formula throughout the entire manuscript.

Materials and methods

7. Give a detailed description of the Study setting and design - First, give us a detailed geographical description of the study setting you carried out this study. What is the justification for the choice of the study setting? Also, second, what is the type of design you employed in this study? Kindly address this.

8. Sampling methods – kindly describe the type of sampling procedures you initiated when collecting the data via online survey platforms. There should be explanation on the study method and, detailed explanations on the methods on the specific tools and procedures use in collecting and analyzing the data should be clarified. Give a justification for the choice of sampling method employed. Kindly address this.

9. Study population and Sampling technique – describe your study population? How did you arrived at reaching out to the population target? Give justification of the choice of these procedures you employed? How did you select them? Give us an adequate and precise procedures you used in getting your population. Discuss in detail both of them with a separate section paragraph for each of them. Insert justification for both differently. Kindly address this.

10. Variable measurements (Outcome variable or Dependent variable) – Where is the outcome variable? A dependent variable is the variable that changes as a result of the independent variable (independent or explanatory variables or factors) manipulation. It's the outcome you're interested in measuring, and it “depends” on your independent variable. In statistically analysis, dependent variables are also called ‘response variables’ (they respond to a change in another variable). Therefore, specify your outcome variable very well and show us how it is going to be measured. Is it a binary outcome variable or what? This section should discuss the measurements of the outcome variable or dependent variable(s). Importantly, Kindly address this.

11. Explanatory variable (factors) or independent variable (factors): Where are the explanatory variable listed? You did not indicate your independent variables you used in this study. I know that you cannot have the dependent variable without the independent variable (factors) when running the statistical analysis. Insert the independent variables and how they are going to be measured (References may be

cited). Define all your Explanatory variables (factors) or Independent variables (factors) and how they were measured in your study. This is very important, as it will affect the process and approach of statistical analysis. Kindly address this.

Statistical analysis –

12. This statement in blue ink [Descriptive statistics were used to characterise the study sample and tests of mean differences (e.g., independent samples *t* tests and analyses of variance [ANOVA]) were used to assess whether value perspective differences existed between consumer and provider groups, as well as to assess value perspective differences as the severity of surgical and medical scenarios increased – Page 9] – is not accurate. Descriptive statistics are not tests of mean differences; rather descriptive statistics allow you to characterize your data based on its properties, and there are four major types of descriptive statistics, which include the following: 1. Measures of Frequency (Count, Percent, and Frequency); 2. Measures of Central Tendency (Mean, Median, and Mode); 3. Measures of Dispersion or Variation (Range, Variance, and Standard Deviation); and 4. Measures of Position (Percentile Ranks, and Quartile Ranks)

Reading materials:

Mishra, P., Pandey, C. M., Singh, U., Keshri, A., & Sabaretnam, M. Selection of Appropriate Statistical Methods for Data Analysis. *Annals of Cardiac Anaesthesia*, *22*(3), 297-301. https://doi.org/10.4103/aca.ACA_248_18.

Ali, Z., & Bhaskar, S. B. (2016). Basic statistical tools in research and data analysis. *Indian Journal of Anaesthesia*, *60*(9), 662-669. https://doi.org/10.4103/0019-5049.190623.

Kim, N., Fischer, A. H., Dyring-Andersen, B., Rosner, B., & Okoye, G. A. (2017). Research Techniques Made Simple: Choosing Appropriate Statistical Methods for Clinical Research. *Journal of Investigative Dermatology*, *137*(10), e173-e178. https://doi.org/10.1016/j.jid.2017. 0 8.007.

13. State and describe the type of statistical analysis suitable for each objective and how they will be analysed in this study.

14. Did you employ the use of parametric statistical tests or non-parametric statistical tests?

15. Did you use the statistical methods in the data analysis? The statistical methods include descriptive statistics (which summarizes data using indexes such as mean and median) and inferential statistics (which draw conclusions from data using statistical tests such as student's t-test).

16. Also, there should be step-by-step description of the analysis by objectives shown in the statistical analysis' sub-section. For instance, objective one on respondents' demographic characteristics was analyzed using univariate analysis (Percentage and frequency including graphs and tables). Objective two measure the associations between the outcome variable and explanatory variables by using the bivariate analysis (Chi-Square or/and Pearson correlation coefficient or non-parametric statistical methods). Objective three measures the relationship influence between the variables and the predictors of the outcome variable on the explanatory factors, when other factors are kept constant (for instance, when using Logistics regression, if and only if the outcome variable is binary, Yes = 1 and No = 0 or the use of non-parametric statistical methods), otherwise, multiple régression analysis, etc.

    This section above must be revised as it is very important. Also, include the models and equations of the bivariate and inferential statistical analysis in your discussion under the section of the statistical analyses. Kindly address this question.

17. This statement in blue is not accurate [Parametric assumptions were evaluated using Kolmogarov-Smirnov and Shapiro-Wilk tests]. Kolmogarov-Smirnov and Shapiro-Wilk tests are not parametric or have parametric assumptions, rather they are non-parametric tests with its assumptions.

18. This statement in blue is not accurate [To examine the association between the variables of interest, univariate analyses using Pearson product-moment correlations were conducted]. It is only Inferential statistics that are involved in examining the associations (use of Chi-square tests) or relationships (Pearson product-moment correlations or Spearman rank).

19. I suggest you put more efforts in understanding the principles of statistical analytical methods. Therefore, kindly explain the each of the statistical methods you want to use and give reasons why you want to use it. For instance, kindly explain how and why you are using the following as you have previously mentioned in the manuscript:

1. Non-parametric statistical test - Kolmogarov-Smirnov test: Assumptions, How and why?
2. Non-parametric statistical test - Shapiro-Wilk test: Assumptions, How and why?
3. Inferential statistical test - Independent sample t-test: Assumptions, How and why?
4. Inferential statistical test - Cohen's d effect size: Assumptions, How and why?
5. Inferential statistical test - One way between-groups ANOVAs: Assumptions, How and why?

20. State and explain the type of analysis you will used to explain each objective of this study

Description of the statistical methods

Kolmogarov-Smirnov test

The Kolmogorov-Smirnov test is used to test the null hypothesis that a set of data comes from a Normal distribution. The Kolmogorov Smirnov test produces test statistics that are used (along with a degrees of freedom parameter) to test for normality. Here we see that the Kolmogorov Smirnov statistic takes value . One-sample K-S test or goodness of fit test was developed by Andrey Nikolayevich Kolmogorov in 1933. Its purpose is to compare the overall shapes of two sample distributions. Two-sample K-S test was developed by Nikolai Smirnov in 1939. Its purpose is to compare one sample to a known statistical distribution. The interpretation of the result, where the p-value, tells you how likely these samples comes from the exact same distribution. When obtained, the p-value can be compared with a threshold call statistical significance (e.g. . 05), if the P-Value is smaller, we can reject the null hypotheses.

Shapiro-Wilk test

The Shapiro–Wilk test can be used to decide whether or not a sample fits a normal distribution, and it is commonly used for small samples. The interpretation of the findings is if the value of the Shapiro-Wilk Test is greater than 0.05, the data is normal, however, if it is below 0.05, the data significantly deviate from a normal distribution. The null hypothesis for a Shapiro Wilk test is that there is no difference between your distribution and a normal distribution. The alternative hypothesis is that there is a difference. If your p value is less than 0.05, which it is, then you reject the null hypothesis and conclude that your data is non-normal.

Independent sample t-test

The independent t-test, also called the two sample t-test, independent-samples t-test or student's t-test, is an inferential statistical test that determines whether there is a statistically significant difference between the means in two unrelated groups. The Independent Samples t Test compares the means of two independent groups in order to determine whether there is statistical evidence that the associated population means are significantly different. The Independent Samples t Test is a parametric test. Paired-samples t tests compare scores on two different variables but for the same group of cases; independent-samples t tests compare scores on the same variable but for two different groups of cases. What are the two types of independent t tests? **The independent samples t-test comes in two different forms: 1.** the standard Student's t-test, which assumes that the variance of the two groups are equal; and 2. Welch's t-test, which is less restrictive compared to the original Student's test. The interpretation of the Independent Samples t Test is If the p-value is less than your significance level (e.g., 0.05), you can reject the null hypothesis.

The difference between the two means is statistically significant. Your sample provides strong enough evidence to conclude that the two population means are not equal.

Cohen's d effect size

There are dozens of measures of effect sizes. The most common effect sizes are Cohen's d and Pearson's r. Cohen's d measures the size of the difference between two groups while Pearson's r measures the strength of the relationship between two variables. Interpreting Cohen's d A commonly used interpretation is to refer to effect sizes as small (d = 0.2), medium (d = 0.5), and large (d = 0.8) based on benchmarks suggested by Cohen (1988). However, these values are arbitrary and should not be interpreted rigidly (Thompson, 2007). What if Cohen's d is greater than 1? A Cohen's d of 1.000 indicates that the means of the two groups differ by 1.000 pooled standard deviation (or one z-score). A Cohen's d of 2.00 indicates that the means of two groups differ by 2.000 pooled standard deviations, and so on. How do you interpret Cohen's d example? A large Cohen's d indicates the mean difference (effect size = signal) is large compared to the variability (noise). For example, if Group A's Mean = 12 and Group B's Mean = 8, and the pooled standard deviation is 2, Cohen's d equals the following: The mean difference is twice the variability.

One way between-groups ANOVAs

ANOVA is categorised into two: 1. between within groups ANOVA; and 2. between groups ANOVA. The within group variation is also called random, unexplained, residual, or variation due to error, and the Residual or within-group variation is assumed to follow the normal, Gaussian, distribution. Also, the between group variation is due to the factor, or explained by the factor. A One-Way Between-Subjects ANOVA compares the means between more than two independent groups, such as comparing the difference between groups A, B and C. If your data only has two groups such as Male/Female or Present/Absent you should consider the Independent-Samples t-Test. In ANOVA, F is the ratio of the error mean square to the model mean square. The P-value is the probability, if the means of the various populations are equal, of a value of F greater than what you observed. By the way, your question is exactly why I dislike terms like P-value*. It doesn't say what it means.

Reading materials

Nahm, F. S. (2016). Nonparametric statistical tests for the continuous data: The basic concept and the practical use. *Korean Journal of Anesthesiology*, *69*(1), 8-14. https://doi.org/10.4097/kjae.2016.69.1.8.

Fagerland, M.W. t-tests, non-parametric tests, and large studies—a paradox of statistical practice?. *BMC Med Res Methodol* **12**, 78 (2012). https://doi.org/10.1186/1471-2288-12-78.

21. Where there missing questionnaires after collation of the questionnaire from the field? If any, how did your account for missing variables or non-responses from the respondents? Kindly look into this.
22. Where is the Ethical approval number assigned to this study research? Please, kindly include the Ethical Approval Reference number.
23. Where is the "Declaration of Helsinki Ethical principles" of this study? What ethical principles did you employed? Did you follow the set of ethical principles. Where is the informed consent form in research? How did you address these factors associated with research such as in ensuring in the protection of participants' rights, ensuring that participants understand the nature and purpose of the research, the risks and benefits of participating, and their rights as participants whether to participate or decline at any time they feel not to. Kindly address this.
24. Was informed consent forms given to the respondents during the data collection via online media outlets you used?

Results

25. There is no table for the demographics for the women respondents. Kindly insert the socio-demographics features of your study respondents.

26. Kindly report the results according to the objectives by using the objective as a theme heading. Show the type of analysis employed and elaborate on the interpretation of the study findings. All the interpretations for the Tables should be included in the results and Table legend should be carefully and appropriately inserted in the main research. Interprete and discuss the relevant and significant variables and make it precise so that it doesn't lose its meaning. Your readers will see the tables with the

results. Kindy address these concerns mentioned. Therefore, kindly revise. For instance, see below:

- Table 1 – Demographic Characteristics of Respondents
- Table 2 – Descriptive statistical analysis
- Table 3 – Inferential statistical analysis (independent t-sample test)
- Table 4 – Non-parametric analysis
- Other relevant tables (if any). Kindly address this.

27. Where is Figure 1?

28. All the findings for this study must be reported according to the objectives of this study.

Discussion

29. The discussion section is one of the final parts of a research paper, in which an author describes, analyzes, and interprets their findings. They explain the significance of those results and tie everything back to the research question(s). The discussion section is where you delve into the meaning, importance, and relevance of your results. It should focus on explaining and evaluating what you found, showing how it relates to your literature review and paper or dissertation topic, and making an argument in support of your overall conclusion. Therefore, let your discussion aspects focused on the interpretation of your findings and relate to literature whether it corroborates or not, with existing studies, alongside with in-citation of recent references. Let each paragraph relates the objective findings. Discuss in assertion or not with study's findings from other studies. Kindly advise.

Conclusion

30. The conclusion should stem from summing up your research paper (by the following steps - restate your research topic, restate the thesis, summarize the main points, state the significance or results, and conclude your thoughts). Always have it in mind that a conclusion is not merely a summary of the main topic(s) covered or a re-statement of your research problem, but a synthesis of key points and, if applicable, where you should recommend new areas for future research.

Strengths of this Study -

31. Where are the Strengths of this Study? Please state them, after the discussion with the sub-Section heading: Strengths and Limitations of this Study after the Discussion Section. Add the limitations of the study after the strengths of the study is included.

Implications of your findings –

32. What are the implications of your findings across other countries with lower prevalence of your outcome of interest? What is the contribution of this study to the existing one, especially in sub-Saharan African or developing countries?

Recommendations

33. Where is the Recommendation for this study? You can add it to the Conclusion section with the sub heading – Conclusion/Recommendations.

References

34. Does this References of this study follow this Journal format? Kindly check and revisit them all.

Editing of the English content of the Manuscript

35. Ask for a Professional English Editors to address all grammatical errors and lexis structure of your manuscript.

Other Comments

36. What is the difference between this present submitted manuscript and this paper of yours with the title: "The Influence of Value Perspectives on Decision-Making in the South African Private Healthcare Sector" by Laubscher, Anchen; URI: http://hdl.handle.net/2263/64862; Date: 2017.
