# Supplementary material for: How value perspectives influence decision-making in the South African private healthcare sector: A cross-sectional comparative study
Source: PLoS One. 2025 Feb 4;20(2):e0316547. doi: 10.1371/journal.pone.0316547 (PMC11793816; doi:10.1371/journal.pone.0316547)
Supplement: Attachment — Submitted filename: REspone to reviews plos 1 2024.11.13.docx [file pone.0316547.s003.docx]

**How value perspectives influence decision-making in the South African private**

**healthcare sector: a cross-sectional comparative study**

**Response to reviewers.**

**Reviwer one**

**Abstract**

1. **The abstract should have an introductory sentence, followed by the objectives and gaps identified. This will make the content of the background under the Abstract section meaningful. Also, include the statistical figures for the results sub-section, especially the significant findings. The formula for the relationship stated under the ‘Result’ sub-section can be written as: VCI = (Outcome ÷ Cost) χ Patient experience instead of expressing it as the Value Care Index (VCI): 𝑉𝐶𝐼 = 𝑜𝑢𝑡𝑐𝑜𝑚𝑒 𝑥 𝑝𝑎𝑡𝑖𝑒𝑛𝑡 𝑒𝑥𝑝𝑒𝑟𝑖𝑒𝑛𝑐𝑒./cost. Kindly rewrite the entire abstract and remove unambiguous information from the abstract. Let it be concise and meaningful.**

Response

We have rewritten the abstract to incorporate the recommendations made. We have unfortunately not been able to include the figures for the significant statistical findings into the abstract results. Because of the multiple comparisons – three factors (cost, clinical outcome, patient experience) by two categories (patients and providers) – incorporating there pushes the word count of the abstract well beyond the allowed 300 words.

**2. The introduction is very short and did not include much vital information on the subject matter. The introduction is the place to review other conclusions on the topic. The introduction section should introduce past findings to those who might not have that expertise. The introduction should have steps such as: introduce the topic. The first phase of the introduction is to tell the reader what the topic is and why it's interesting or important by describing the background; establishing the research problem; specifying the objective(s); and mapping out the paper. Also, remember that there are general phases that are associated with writing an introduction: stating the intent of the study, outlining the key characteristics of the study, describing important results, and giving a brief overview of the structure of the paper. Note that, the introduction of this study should follow the ‘Funnel Shape’ narrative method to scope and review existing studies across the worldwide and the study setting. This will illustrate the trends, pattens and prevalence rates of the outcome of interests of this study. Kindly revise.**

**3. Where are the gaps identified in this study? What has the previous studies have mentioned as regards the outcome(s) of this study? What is the current study saying on the current situation of your study outcome(s)? Always remember that a gap in a research study is an unanswered question or unresolved problem in a field, which reflects a lack of existing research in that space or it can exist when there is a concept or new idea that hasn't been studied at all, when the existing research is outdated or insufficient, or when there is a disagreement, contextual or methodological issue among the researchers. A gap in a research study indicates an opportunity for further investigation and contribution to the scientific knowledge. Kindly revise.**

**4. Where are the main objective of this study? Where are the specific objectives of this study? Kindly state them clearly.**

**Reponses to 2, 3 and 4.**

Thank you, we have expanded the introduction to consider these recommendations.

**5. The Keywords are so funny in nature…you have to follow the Journal format of presenting Keywords for your study. However, I may suggest the Keywords should be: Clinical outcome, patient experience, healthcare, South Africa, value perspectives,**

Response

Thank you we have made use of these key words

**6. Kindly re-write the formula for the relationship stated throughout the entire manuscript as: VCI = (Outcome ÷ Cost) χ Patient experience Kindly revise the formula throughout the entire manuscript.**

Response

Thank you – we have made this change.

**7. Give a detailed description of the Study setting and design - First, give us a detailed geographical description of the study setting you carried out this study. What is the justification for the choice of the study setting? Also, second, what is the type of design you employed in this study? Kindly address this.**

Response:

This information was provided in the materials and methods section. The text reads as follows:

*“This study was a non-experimental, cross-sectional, comparative study between healthcare consumer and provider groups within the private healthcare sector in South Africa. The industry in which the study was conducted was the South African healthcare sector, specifically, the private sector where payment is made for medical services rendered. Clinicians are not employed by private healthcare institutions and operate as independent practitioners in these facilities, as dictated by the Health Professionals Council of South Africa (HPCSA). Patients are either insured by subscribing to and purchasing of medical aid cover or they pay for private medical care by means of cash at point of care.”*

**8. Sampling methods – kindly describe the type of sampling procedures you initiated when collecting the data via online survey platforms. There should be explanation on the study method and, detailed explanations on the methods on the specific tools and procedures use in collecting and analyzing the data should be clarified. Give a justification for the choice of sampling method employed. Kindly address this.**

Response:

This information was provided in the second part of the materials and methods section. The text reads as follows:

*“A non-probability sampling technique was used to collect data and included convenience,*

*purposive and snowball sampling strategies [6]. The first author (AL) utilised her professional and personal network to collect data from the two sample groups using various social media platforms. The purposive strategy was employed to ensure a high number of clinician participants across a wide spectrum of subspecialties and registration categories with the HPCSA. Included in the sample were doctors working in both administrative and clinical capacities. Snowball sampling was used to leverage the networks available to the researcher. Participants were asked to distribute the survey to other potentially willing individuals both when they received an invitation and following completion of the survey.”*

**9. Study population and Sampling technique – describe your study population? How did you arrived at reaching out to the population target? Give justification of the choice of these procedures you employed? How did you select them? Give us an adequate and precise procedures you used in getting your population. Discuss in detail both of them with a separate section paragraph for each of them. Insert justification for both differently. Kindly address this.**

Response:

This information was provided in the materials and methods. It reads as follows:

*This study was a non-experimental, cross-sectional, comparative study between healthcare consumer and provider groups within the private healthcare sector in South Africa. The industry in which the study was conducted was the South African healthcare sector, …*

*A non-probability sampling technique was used to collect data and included convenience, purposive and snowball sampling strategies [6]. The first author (AL) utilised her professional and personal network to collect data from the two sample groups using various social media platforms. The purposive strategy was employed to ensure a high number of clinician participants across a wide spectrum of subspecialties and registration categories with the HPCSA. Included in the sample were doctors working in both administrative and clinical capacities….*

*… Data were collected through a self-administered online survey, the Value Perspectives Survey, using the survey platform Qualtrics (full survey available in Appendix 1) and were collect from 1 to 30 September 2017. The survey link directed the participant to a cover letter after which consent was obtained. Participants were then directed to either the patient or doctor section of the survey. To ensure a between-subjects design, a participant could not compete the survey from both consumer and provider perspectives. Should a participant work in the healthcare industry as a healthcare worker in any category other than that of a doctor, they were required to complete the survey from a patient perspective.*

**10. Variable measurements (Outcome variable or Dependent variable) – Where is the outcome variable? A dependent variable is the variable that changes as a result of the independent variable (independent or explanatory variables or factors) manipulation. It's the outcome you're interested in measuring, and it “depends” on your independent variable. In statistically analysis, dependent variables are also called ‘response variables’ (they respond to a change in another variable). Therefore, specify your outcome variable very well and show us how it is going to be measured. Is it a binary outcome variable or what? This section should discuss the measurements of the outcome variable or dependent variable(s). Importantly, Kindly address this.**

Response

The outcome was specifically provided in the materials and methods section and formed the basis of the analysis we conducted. It read as follows:

*“After obtaining basic demographic information the survey presented clinical scenarios aimed at examining the participants’ value perspectives. They were asked to distribute 100 points between clinical outcomes, event cost, and patient experience according to their perceived relative importance.”*

To make this clearer to the reader we have added clarifying information. The section now reads as follows:

*“After obtaining basic demographic information the survey presented clinical scenarios aimed at examining the participants’ value perspectives – the primary focus of this study. They were asked to distribute 100 points between clinical outcomes, event cost, and patient experience according to their perceived relative importance.* ***For example, a participant evaluating a clinical scenario where they were undergoing surgery for a hernia repair could decide to allocate 30 of the available 100 points to clinical outcome, 50 points to event cost, and 20 to patient experience****.”*

**11. Explanatory variable (factors) or independent variable (factors): Where are the explanatory variable listed? You did not indicate your independent variables you used in this study. I know that you cannot have the dependent variable without the independent variable (factors) when running the statistical analysis. Insert the independent variables and how they are going to be measured. Define all your Explanatory variables (factors) or Independent variables (factors) and how they were measured in your study. This is very important, as it will affect the process and approach of statistical analysis. Kindly address this.**

Response:

This information was provided in multiple places in both the introduction, and formally in the materials and methods section. The two independent variables, or groups of interest, were healthcare consumers and healthcare providers. This was stated in the following sections:

Introduction

- *“Further to exploring the role of Patient’s Experience in Value of Care, the purpose of the study is to obtain a deeper understanding of the* ***differences in value perspectives between healthcare consumer and provider groups****”*
- *“This study, a quantitative review of primary data, aims to gain insight into* ***the value perspectives of consumers and providers*** *in the South African private healthcare context and to examine if these* ***value perspectives differ between the two groups****. Both providers and consumers from the South African private sector were included in this study.”*
- *“The study explored the differences between the relative importance of the three factors (Clinical Outcome, Cost of Clinical Event, and Patient’s Experience) that make up the* ***Value of Care for both consumers and providers****, in general and specifically in terms of medical and surgical procedures.”*
- *“The objective of this study was to … understand how relative importance between the three factors might differ between* ***consumer and provider groups*** *as the clinical severity of the scenarios changed.”*

Material and methods

- *“This study was a non-experimental, cross-sectional, comparative study between* ***healthcare consumer and provider groups*** *within the private healthcare sector in South Africa.”*

Data analysis

- “Descriptive statistics were used to characterise the study sample and tests of mean differences (e.g., independent samples *t* tests and analyses of variance [ANOVA]) were used to assess whether value perspective differences **existed between consumer and provider groups**”

The definition of the consumer and provider groups were provided in the materials and methods section.

**12. This statement in blue ink [Descriptive statistics were used to characterise the study sample and tests of mean differences (e.g., independent samples t tests and analyses of variance [ANOVA]) were used to assess whether value perspective differences existed between consumer and provider groups, as well as to assess value perspective differences as the severity of surgical and medical scenarios increased – Page 9] – is not accurate. Descriptive statistics are not tests of mean differences; rather descriptive statistics allow you to characterize your data based on its properties, and there are four major types of descriptive statistics, which include the following: 1. Measures of Frequency (Count, Percent, and Frequency); 2. Measures of Central Tendency (Mean, Median, and Mode); 3. Measures of Dispersion or Variation (Range, Variance, and Standard Deviation); and 4. Measures of Position (Percentile Ranks, and Quartile Ranks)**

Response

Thank you for this comment.

Our sentence read as follows*: “Descriptive statistics were used to characterise the study sample* ***and*** *tests of mean differences (e.g., independent samples t tests and analyses of variance [ANOVA]) were used to assess whether value perspective differences existed between consumer and provider groups, as well as to assess value perspective differences as the severity of surgical and medical scenarios increased.”*

As the sentence says, descriptive statistics were used to characterize the study sample.

*“Descriptive statistics were used to characterise the study sample.”*

And then tests of mean difference were used to assess the differences between the two groups.

***“…and*** *tests of mean differences (e.g., independent samples t tests and analyses of variance [ANOVA]) were used to assess whether value* *perspective differences existed.”*

This formulation is standard when describing basic statistical methods.

**13. State and describe the type of statistical analysis suitable for each objective and how they will be analysed in this study.**

Response:

We refer the reviewer to the section of the manuscript titled “Data analysis”, and specifically the section that reads:

*“ … tests of mean differences (e.g., independent samples t tests and analyses of variance [ANOVA]) were used to assess whether value perspective differences existed between consumer and provider groups, as well as to assess value perspective differences as the severity of surgical and medical scenarios increased.”*

*“… To examine the association between the variables of interest, univariate analyses using Pearson product-moment correlations were conducted. Independent samples t-tests were conducted to assess whether provider and consumer groups weighted value perspectives differently across the general value baseline and the surgical, and medical scenarios. Cohen’s d effect sizes were calculated to determine practical differences between the weighted perspectives. One way between-groups ANOVAs were conducted to determine whether differences existed in value perspectives across severity of surgical and medical scenarios for both consumer and provider groups.”*

These specifically note the outcome being analyzed, as well as the test being used.

**14. Did you employ the use of parametric statistical tests or non-parametric statistical tests?**

Response:

We refer the reviewer to the section of the manuscript titled “Data analysis” that lists the tests used. It is not standard to note that t-tests or ANOVA tests are parametric tests. However, as the text states, we did evaluate the parametric assumptions of the test by way of the Kolmogarov-Smirnov and Shapiro-Wilk tests.

15. **Did you use the statistical methods in the data analysis? The statistical methods include descriptive statistics (which summarizes data using indexes such as mean and median) and inferential statistics (which draw conclusions from data using statistical tests such as student's t-test).**

Response:

Yes, we used the statistical methods as we listed them in the data analysis. Please review our response to Question 12. This should clarity any misunderstanding on the methodology we followed.

16. **Also, there should be step-by-step description of the analysis by objectives shown in the statistical analysis’ sub-section. For instance, objective one on respondents’ demographic characteristics was analyzed using univariate analysis (Percentage and frequency including graphs and tables). Objective two measure the associations between the outcome variable and explanatory variables by using the bivariate analysis (Chi-Square or/and Pearson correlation coefficient or non-parametric statistical methods). Objective three measures the relationship influence between the variables and the predictors of the outcome variable on the explanatory factors, when other factors are kept constant (for instance, when using Logistics regression, if and only if the outcome variable is binary, Yes = 1 and No = 0 or the use of non-parametric statistical methods), otherwise, multiple régression analysis, etc.**

Response:

Please see our response to Question 10 where we highlight the outcome variable. Based on this, the data analysis section does specify each step of the analyses that we have undertaken.

- We statistically described the study population characteristics.
  - The data analysis section reads: “*Descriptive statistics were used to characterise the study sample*”
- We compared the difference in value perspectives between the consumer and provider groups
  - The data analysis section reads: “*tests of mean differences (e.g., independent samples t tests and analyses of variance [ANOVA]) were used to assess whether value* *perspective differences existed between consumer and provider groups, as well as to assess value perspective differences as the severity of surgical and medical scenarios increased*.”
- We evaluated the association between the two groups
  - The data analysis section reads: “*To examine the association between the variables of interest, univariate analyses using Pearson product-moment correlations were conducted. Independent samples t-tests were conducted to assess whether provider and consumer groups weighted value perspectives differently across the general value baseline and the surgical, and medical scenarios. Cohen’s d effect sizes were calculated to determine practical differences between the weighted perspectives.”*
- We should to determine whether there was a difference in value perspectives across surgical and medical scenarios.
  - The data analysis section reads: “*One way between-groups ANOVAs were conducted to determine whether differences existed in value perspectives across severity of surgical and medical scenarios for both consumer and provider groups.”*

**16 b. This section above must be revised as it is very important. Also, include the models and equations of the bivariate and inferential statistical analysis in your discussion under the section of the statistical analyses. Kindly address this question.**

Response:

As noted above we do not believe that it is necessary to revise this section as each step of our analysis has been clearly explained in the data analysis section. Further, we do not believe that it is appropriate to include the statistical formulas for basic statistical tests in this manuscript.

17. **This statement in blue is not accurate [Parametric assumptions were evaluated using Kolmogarov-Smirnov and Shapiro-Wilk tests]. Kolmogarov-Smirnov and Shapiro-Wilk tests are not parametric or have parametric assumptions, rather they are non-parametric tests with its assumptions.**

Response:

The reviewer seems to have misunderstood this sentence. To say that “Parametric assumptions were evaluated using Kolmogarov-Smirnov and Shapiro-Wilk tests” is a standard formulation to say that we use the two tests to determine whether the underlying data was normally distributed or not. As we applied parametric tests (t-test and ANOVA) it was important to test the underlying data to validate our use of these two tests.

18. **This statement in blue is not accurate [To examine the association between the variables of interest, univariate analyses using Pearson product-moment correlations were conducted]. It is only Inferential statistics that are involved in examining the associations (use of Chi-square tests) or relationships (Pearson product-moment correlations or Spearman rank).**

Response:

The reviewer seems to have misunderstood this sentence or seems to assign statistical significance to the word “association” as opposed to “relationships”.

The appropriate test by which evaluates the measure of the strength of a linear association between two variables is the Pearson product-moment correlation coefficient. Please note in the references below that the word association is used interchangeably with relationship.

- (<https://statistics.laerd.com/statistical-guides/pearson-correlation-coefficient-statistical-guide.php>)
- <https://en.wikipedia.org/wiki/Pearson_correlation_coefficient>
- https://mathresearch.utsa.edu/wiki/index.php?title=Univariate,_Bivariate,_Correlation_and_Causation#Pearson.27s_product-moment_coefficient

It is therefore correct to state that we examined the association between the variables of interest using the Pearson product-moment correlations. It would be incorrect to say that we used inferential statistics as inferential statistics are testing to determine the probability that there is a difference between two groups and do not seek to describe the relationship or association between the two groups.

19. **I suggest you put more efforts in understanding the principles of statistical analytical methods. Therefore, kindly explain the each of the statistical methods you want to use and give reasons why you want to use it. For instance, kindly explain how and why you are using the following as you have previously mentioned in the manuscript:**

- **Non-parametric statistical test - Kolmogarov-Smirnov test: Assumptions, How and why?**
- **Non-parametric statistical test - Shapiro-Wilk test: Assumptions, How and why?**
- **Inferential statistical test - Independent sample t-test: Assumptions, How and why?**
- **Inferential statistical test - Cohen’s d effect size: Assumptions, How and why?**
- **Inferential statistical test - One way between-groups ANOVAs: Assumptions, How and why**

Response:

As demonstrated, our statistical methods are sound and appropriately applied. It is highly nonstandard to provide the assumptions to the level being requested here. Further, by virtue of the tests being used, the assumptions being made are clear. Where required, we have tested these assumptions.

20. **State and explain the type of analysis you will used to explain each objective of this study**

Response:

Please see our response above.

21. **Where there missing questionnaires after collation of the questionnaire from the field? If any, how did your account for missing variables or non-responses from the respondents? Kindly look into this.**

Response:

As noted in the materials and methods section, “*Data were collected through a self-administered online survey*.” Therefore, there could not be any missing questionnaires.

22. **Where is the Ethical approval number assigned to this study research? Please, kindly include the Ethical Approval Reference number.**

Response:

We have added this to the manuscript. The text now reads as follows:

*“Ethical clearance was obtained from the Gordon Institute of Business Science Research Ethics Committee and the University of Pretoria Human Research Ethics Committee (Temp2017-00651, 18/07/2017) before any research was conducted.”*

23. **Where is the “Declaration of Helsinki Ethical principles” of this study? What ethical principles did you employed? Did you follow the set of ethical principles. Where is the informed consent form in research? How did you address these factors associated with research such as in ensuring in the protection of participants’ rights, ensuring that participants understand the nature and purpose of the research, the risks and benefits of participating, and their rights as participants whether to participate or decline at any time they feel not to. Kindly address this.**

Response:

The ethical principles by which the study was conducted are contained in the section labeled “Ethical considerations.”. All studies approved by the ethics board must conform to a range of ethical principles. These include the Helsinki Declaration, Good Clinical Research Practice, as well as local research ethics guidelines. It is not standard to list the declarations or principles in a research paper. However, we have added to the ethics section which now reads as follows:

*“ Ethical considerations*

*Data collection, specifically in the healthcare sector, requires ethical practises, including respect for the participants, as well as transparency of what is being researched and confidentiality of data. Ethical clearance was obtained from the Gordon Institute of Business Science Research Ethics Committee and the University of Pretoria Human Research Ethics Committee (Temp2017-00651, 18/07/2017) before any research was conducted.*

*Participants were made aware that the results may be presented publicly and published in an academic journal. Furthermore, the results of the survey were kept private, and confidentiality was upheld throughout. Data were analysed and reported on in an anonymised, aggregated manner and the researcher ensured that participants could by no means be identified as no identifying information (name, ID etc.) was requested in the survey.*

*Individuals wanting to participate in this study needed to do so voluntarily. After receiving information on the nature and objective of the study, participation required indicating consent on the electronic questionnaire, which stipulated the voluntary nature of the study. There were no benefits or harm to participating in this study and participants could stop at any time without negative consequences.*

*When conducting research involving humans it is important to note ethical concerns that may arise during the execution of the research study. Throughout the research study the principal researcher ensured that ethical standards were upheld.”*

The specifics relating to informed consent can be found in the section labeled “Ethical considerations.” The section reads as follows: *“Individuals wanting to participate needed to do so voluntarily, indicating consent in writing on the electronic survey.”*

Additional information on the methodology can be found in the materials and method section that reads as follows:

“*Data were collected through a self-administered online survey, the Value Perspectives Survey, using the survey platform Qualtrics (full survey available in Appendix 1) and were collect from 1 to 30 September 2017. The survey link directed the participant to a cover letter after which consent was obtained. Participants were then directed to either the patient or doctor section of the survey. To ensure a between-subjects design, a participant could not compete the survey from both consumer and provider perspectives. Should a participant work in the healthcare industry as a healthcare worker in any category other than that of a doctor, they were required to complete the survey from a patient perspective.”*

24. **Was informed consent forms given to the respondents during the data collection via online media outlets you used?**

Response:

As stated in the materials and methods – *“The survey link directed the participant to a cover letter after which consent was obtained. Participants were then directed to either the patient or doctor section of the survey.”*

25. **There is no table for the demographics for the women respondents. Kindly insert the socio-demographics features of your study respondents.**

Response:

We did not report any of the demographics in table form as the data in this section is very scant and best presented in text form.

We detailed the number of female respondents in the first line of the demographics section. The text reads as follows*:*

*“The study included a total of 1043 participants between 19 to 88 years with a mean age of 44 years (standard deviation [SD] 11.8) of which 58% (n = 605) were female.”*

26. **Kindly report the results according to the objectives by using the objective as a theme heading. Show the type of analysis employed and elaborate on the interpretation of the study findings. All the interpretations for the Tables should be included in the results and Table legend should be carefully and appropriately inserted in the main research. Interprete and discuss the relevant and significant variables and make it precise so that it doesn’t lose its meaning. Your readers will see the tables with the results. Kindy address these concerns mentioned. Therefore, kindly revise. For instance, see below:**

**- Table 1 – Demographic Characteristics of Respondents**

**- Table 2 – Descriptive statistical analysis**

**- Table 3 – Inferential statistical analysis (independent t-sample test)**

**- Table 4 – Non-parametric analysis**

**- Other relevant tables (if any). Kindly address this.**

Response:

Thank you for this comment.

- The results section of the manuscript is now structured as per the aims of the study and the data analysis.
- We have added additional headings to assist the reader in navigating the findings.
- We have elected not to add the demographic details in table form as we believe the data in this section is very scant and thus is best managed in text form.
- We have left the discussion of the relevant findings in the discussion section and not in the results section.
- We have included a careful and appropriate summary of each table’s findings in the results section.

27. **Where is Figure 1?**

Response:

As per the instructions to authors, figures were not to be included in the main manuscript but were loaded separately. To access the figure please review the instructions to reviewers.

28. **All the findings for this study must be reported according to the objectives of this study.**

Response:

Thank you, we have done so.

29. **The discussion section is one of the final parts of a research paper, in which an author describes, analyzes, and interprets their findings. They explain the significance of those results and tie everything back to the research question(s). The discussion section is where you delve into the meaning, importance, and relevance of your results. It should focus on explaining and evaluating what you found, showing how it relates to your literature review and paper or dissertation topic, and making an argument in support of your overall conclusion. Therefore, let your discussion aspects focused on the interpretation of your findings and relate to literature whether it corroborates or not, with existing studies, alongside with in-citation of recent references. Let each paragraph relates the objective findings. Discuss in assertion or not with study’s findings from other studies. Kindly advise.**

Response:

Thank you, we have done so, had added additional information to the discussion, and have added subheadings to provide structure to the discussion.

**Conclusion**

30. **The conclusion should stem from summing up your research paper (by the following steps - restate your research topic, restate the thesis, summarize the main points, state the significance or results, and conclude your thoughts). Always have it in mind that a conclusion is not merely a summary of the main topic(s) covered or a re-statement of your research problem, but a synthesis of key points and, if applicable, where you should recommend new areas for future research.**

Response:

Thank you, we have modified the conclusion to consider these recommendations.

31. **Where are the Strengths of this Study? Please state them, after the discussion with the sub-Section heading: Strengths and Limitations of this Study after the Discussion Section. Add the limitations of the study after the strengths of the study is included.**

Response:

Thank you, we have added this to the manuscript. It reads as follows:

*“Strengths*

*The observational nature of this study gives its findings broad applicability within the South Africa healthcare context. Its large sample size relative to the South African healthcare ensures that its findings will have significant relevance and external validity.*

*Limitations*

*This study is limited in that the consumer sample may have included other healthcare practitioners such as nurses, paramedics, pharmacists, physiotherapists, occupational therapists, and dieticians, for example. However, as these healthcare practitioners will all at one stage be healthcare consumers, this is likely to increase the generalisability of the study findings. Further, we were not able to explore the factors influencing value assignment in each group, thereby limiting the explanatory power of the study.”*

32. **Implications of your findings – What are the implications of your findings across other countries with lower prevalence of your outcome of interest? What is the contribution of this study to the existing one, especially in sub-Saharan African or developing countries?**
33. **Where is the Recommendation for this study? You can add it to the Conclusion section with the sub heading – Conclusion/Recommendations.**

Response: As noted in the manuscript, this study is not examining the prevalence of any disease or outcome. It is asking participants to allocate value to the three parts of the value equations – clinical outcome, cost and patient experience. Thus, there can be no implication relative to any lower prevalence.

We have added additional text describing the possible implications of the study in other developing countries and have highlighted this as an area for additional study. The section now reads as follows:

*“It would be of value to repeat a similar study within different health care and socioeconomic contexts. It is likely that the perspectives of both health care providers and consumers would change based on the socioeconomic development level of their country as well as the availability and stability of that country’s health care system.”*

**34. Does this References of this study follow this Journal format? Kindly check and revisit them all.**

Response:

Yes. We have used the Plos-1 standard in the reference manager for all references

35. **Editing of the English content of the Manuscript . Ask for a Professional English Editors to address all grammatical errors and lexis structure of your manuscript.**

Response:

This manuscript was reviewed by a professional editor before its submission.

36. **What is the difference between this present submitted manuscript and this paper of yours with the title: “The Influence of Value Perspectives on Decision-Making in the South African Private Healthcare Sector”**

Response:

As noted in the declarations, this is a manuscript based on the original thesis that was submitted for degree purposes by the first author. The thesis is currently only available in the University thesis archive.

**Reviewer 2**

1. **The concept of integrating patient experience, clinical outcomes, and cost to measure healthcare value, as proposed in this study through the Value Care Index (VCI), aligns closely with the well-established Triple Aim framework by the Institute for Healthcare Improvement (IHI). Introduced by Berwick et al. in 2008 (Berwick DM, Nolan TW, Whittington J. The Triple Aim: Care, Health, and Cost. Health Aff (Millwood). 2008;27(3):759-69), the IHI Triple Aim already emphasizes the balance between population health outcomes, per capita costs, and patient experience.

   Given that similar work has been done, I suggest the authors clarify how their approach offers novel insights beyond the Triple Aim. It would also be helpful for the authors to address the existing body of research and explain how their VCI differs from or improves upon this widely recognized framework.**

Response:

Our introduction identifies Berwick as the originator of this framework and we use the first three paragraphs to explore their work, as well as contributions made by other authors on this topic (i.e., Damber and Porter). Our analysis specifically aims to understand the relative importance of Patient experience in relation to the other factors – outcome and cost, and then how this relative important difference between consumer and provider groups. The text reads as follows:

“*The study therefore aimed to determine the need to add Patient’s Experience to the healthcare value equation as originally described, to understand the relative importance of this additional factor in relation to other factors, and to examine how this relative importance might differ between consumer and provider groups.*

*Further to exploring the role of Patient’s Experience in Value of Care, the purpose of the study is to obtain a deeper understanding of the differences in value perspectives between healthcare consumer and provider groups. …*

*This study, a quantitative review of primary data, aims to gain insight into the value perspectives of consumers and providers in the South African private healthcare context and*

*to examine if these value perspectives differ between the two groups.”*

**2. Feedback on Introduction Structure:

The paragraph that outlines the study's objectives is important, but it could be better placed after a more detailed discussion of the existing literature and research gap. I recommend:

Expanding the background on patient experience, clinical outcomes, and event cost with appropriate citations.
Clarifying the research gap by explaining why the relationship between patient experience and value is less understood.
Moving the research objectives to a separate paragraph at the end of the introduction, which will provide a clearer transition from the background to the aims of the study.**

Response:

Thank you, we have restructured the introduction as suggested.

**3. While the manuscript is well-structured and covers a critical topic, I would like to bring your attention to a minor grammatical issue. On page 6, the sentence reads: “Data were collect from 1 to 30 September 2017.” The correct phrase should be: “Data were collected from 1 to 30 September 2017.”**

Response:

Thank you, we have corrected this error.

**4. While the "Materials and Methods" section is thorough and detailed, which is commendable, the "Introduction" and "Literature Survey" sections are comparatively underdeveloped. To set the context of the study more effectively, the introduction should include a more comprehensive literature review that evaluates similar works done in the area. This would help in framing the research gap more explicitly and support the study's objectives.**

**I suggest the following improvements:

Expand the literature review: Include more studies that explore the relationship between patient experience, clinical outcomes, and event costs in healthcare, particularly in similar contexts. This would enhance the academic rigor and provide a stronger foundation for the study.
Clarify the research gap: Explicitly state what existing studies have missed and why this study is essential in the South African healthcare context.
Condense some parts of the Materials and Methods: While the detailed methodology is valuable, some minor adjustments to condense certain descriptions may help balance the overall structure of the paper.**

Response:

Thank you for these comments. We have expanded the introduction and enhanced the literature review. However, we have unfortunately expanded the methods and materials section to address the queries raised by the first reviewer. We trust that this will be acceptable.

**Review Comment:**

**1. While it is understood that the questionnaire has already been administered, the choice of clinical scenarios in Table 1 raises some concerns regarding how non-healthcare professionals (non-HCPs) would relate to and comprehend the descriptions, particularly for complex procedures they may not have experienced (e.g., "liver cancer that has spread" or "severe shock as a complication of a heart attack"). This could have affected the validity of their responses.**

**To address this, I suggest the authors:

Provide a justification for the selection of these specific clinical examples. Why were these particular scenarios chosen, and on what basis were they considered relatable to non-HCPs?
Clarify the rationale for expecting non-HCPs to rate their expectations for procedures they may not have personal experience with.
Acknowledge the potential limitation this introduces and discuss how it may impact the interpretation of the results. For instance, did the authors anticipate this issue and take any measures to mitigate it?**

Response:

The structure of the three questions in the medical and surgical severity questions are designed to be graded from minor, to moderate and then to severe. The progressive nature of the disease severity was selected to allow the respondent to understand that in the scenario they become progressively sicker. The second important factor to note was that the place where care was being provided also changed. Minor – day ward, moderate – general ward, severe – intensive care.

We do not believe that it would be required for the respondent to have experienced these events to be able to give a reliable assessment of their assessment of the value outcomes. Just as it is unlikely that they would have experienced a heart attack or liver cancer, it is also very likely that most do not have high blood sugar, have not been admitted to hospital for pneumonia, or any of the other scenarios. What is clear, however, is that each of the scenarios is more severe and, in the major severity – where admission is into ICU, possibly life threatening. The surgical cases progress from skin flap to groin hernia to cancer. And the medical cases progress from high blood sugar, to pneumonia, to heart attack and sepsis.

To address these points, we have added additional details into the methods and material section which now reads as follows:

*“Scenarios were selected to include conditions that could be experienced by patients and encountered by clinicians across different age groups and genders. The scenarios used are described in Table 1.*

*The scenario questions were designed to indicate the progressive severity of the disease process. For surgical cases – from minor skin flap, to groin hernia, to cancer; and for medical cases – from high blood sugar, to pneumonia, to heart attack with septic shock. Further, the location where case was to be provided was also graded to indicate progression in disease severity – from day ward, to general ward, to intensive care unit.”*

We had added a limitation into the limitations section which reads as follows:

*“An additional limitation is that respondents, particularly non-healthcare practitioners, may not have personally experienced the clinical scenarios used in the questionnaire. However, the severity of the different categories was not specifically linked to the clinical scenarios described, but more broadly to the severity of those scenarios. The difference between being treated in a day ward, so compared to a general ward or intensive care would be apparent to non-healthcare professionals.”*

2. **The "Data Analysis" section provides a good overview of the statistical techniques employed. However, I recommend a few enhancements for clarity and comprehensiveness:

   Clarify alignment with research questions: While you mention the statistical tests used, it would strengthen the section to explicitly link these tests to the specific research questions or hypotheses they were designed to address (e.g., which tests were used to assess differences in value perspectives across severity levels).**

Response

Thank you for this comment. We have made additions to the text that now reads as follows:

*“Descriptive statistics were used to characterise the study sample.*

*T**ests of mean differences i.e, independent samples t test, was used to assess whether value perspective differences existed between consumer and provider groups and analyses of variance [ANOVA] tests were used to assess value perspective differences such as the severity of surgical and medical scenarios increased.*

*Parametric assumptions were evaluated using* *Kolmogarov-Smirnov and Shapiro-Wilk tests.*

*To examine the association between these variables of interest, univariate analyses using Pearson product-moment correlations were conducted.*

*Independent samples t-tests were conducted to assess whether provider and consumer groups weighted value perspectives differently across the general value baseline and the surgical, and medical scenarios. Cohen’s d effect sizes were calculated to determine practical differences between the weighted perspectives.*

*One way between-groups ANOVAs were conducted to determine whether differences existed in value perspectives across severity of surgical and medical scenarios for both consumer and provider groups. “*

3. **Missing data handling: Please clarify if any missing data were present in the dataset and how this was handled in the analysis (e.g., listwise deletion, imputation).**

Response: Where data was missing for specific fields, these patients were excluded from that analysis. No data imputation was performed. These points have been added to the manuscript.

4. **Effect size interpretation: You mention Cohen’s d for effect size; it might be useful to briefly state how the effect sizes were interpreted (small, medium, large), as this will help readers understand the practical significance of the findings.**

Response:

We have added the following to the methods section and have added Cohen’s d and eta squared to the appropriate tables:

*“Cohen’s d effect sizes were calculated to determine practical differences between the weighted perspectives between provider and consumer groups, with effect size classified as follows: small (d = 0.2), medium (d = 0.5) and large (≥ 0.8). One way between-groups ANOVA tests were conducted to determine whether differences existed in value perspectives across severity of surgical and medical scenarios for both consumer and provider groups. The effect size for these analyses using ANOVA were calculated using eta squared (η^2)^, with effect size classified as follows: small (η^2^= 0.01), medium (η^2^ = 0.06) and large (η^2^ = 0.14. effect sizes were calculated to determine practical differences between the weighted perspectives with effect size was classified as follows: small (d = 0.2), medium (d = 0.5) and large (≥ 0.8).”*

5. **Assumption checks: The section on parametric assumptions (Kolmogorov-Smirnov and Shapiro-Wilk tests) is helpful. However, consider noting how any violations of these assumptions were handled (e.g., transformations or non-parametric alternatives).**

Response – for the most part the data was found to be normally distributed and parametric techniques were employed. We have added this to the results section.

6. **The proposed Value Care Index (VCI) offers a compelling way to incorporate patient experience into the healthcare value equation, and the consistent valuation of patient experience across scenarios supports this idea. However, as this is a significant claim, it might benefit from additional refinement and validation using a broader set of data or more diverse clinical contexts. I suggest the following:**

   **Consider more data for validation: While the current data provides a strong starting point, further validation across different healthcare settings or additional patient groups could lend more credibility to the equation. This could be addressed as a future research direction.
   Discuss limitations: Acknowledging the potential limitations of applying the VCI broadly and proposing future studies to refine the coefficients and weightings would show a balanced view without undermining the innovation of this idea.**

Response: We have addressed these points in the limitations section as requested.

Limitations

*“… Further, we were not able to explore the factors influencing value assignment in each group, thereby limiting the explanatory power of the study. Our findings are limited in that they relate to the specific population we studied. Therefore, care should be taken when interpreting the coefficients and weightings reported in this study. Similar studies conducted in different populations would assist in addressing this limitation.”*

Future studies

“*It would be of value to repeat a similar study within different health care and socioeconomic contexts. It is likely that the perspectives of both health care providers and consumers would change based on the socioeconomic development level of their country as well as the availability and stability of that country’s health care system. As such, our findings are specific to the South African private healthcare context. Further validation in other healthcare setting or patient groups would add greater credibility to these findings.”*

Review Comment:

7. **In addition to the limitations already mentioned, I recommend that the authors address the geographical limitations of the study. The findings, particularly those related to value perspectives in healthcare, may be specific to the South African private healthcare context and may not be directly applicable to other healthcare systems globally. Given that healthcare values can vary by country and region, acknowledging this limitation will provide a clearer understanding of how widely the results can be generalized. I also suggest discussing the potential differences in value perspectives between healthcare systems globally and how these might impact the applicability of the proposed Value Care Index (VCI).**

Response: We have added these points to the limitations section, which now reads as follows:

*“**It would be of value to repeat a similar study within different health care and socioeconomic contexts. It is likely that the perspectives of both health care providers and consumers would change based on the socioeconomic development level of their country as well as the availability and stability of that country’s health care system. As such, our findings are specific to the South African private healthcare context. Further validation in other healthcare setting or patient groups would add greater credibility to these findings.”*

8. **The section on aligning healthcare around the goal of providing patients with the highest possible value of care is crucial, especially in the South African private healthcare context. However, I believe it can be improved by more clearly highlighting the study's specific findings and their implications. For example, the fact that patient experience consistently holds a higher perceived value than event cost across various scenarios should be emphasized. Additionally, the differences in value perception between consumers and providers regarding clinical outcomes, patient experience, and costs should be made more explicit, as these are key insights that can inform value-driven healthcare strategies.
   I recommend revising the paragraph to focus more on the findings and their relevance to healthcare leaders in South Africa. This will make the study’s contributions clearer and more impactful.**

Response:

We have taken these points into account. We have added the following to the discussion section:

“*An interesting phenomenon was demonstrated in the array of surgical value perspectives in terms of event cost and clinical outcome where the highest value across scenarios was given to surgical cost in the mild severity example but the lowest given in the moderate severity scenario. This was different from the medical array of scenarios where the greatest value was given to the most severe scenario. Similarly, the highest value across scenarios for surgical clinical outcome was in the moderate severity category with the second highest in the severe category. This was again different from the result obtained in the medical array of scenarios. Of further importance was the finding that patient experience consistently held a greater perceived value as compared to event cost. This highlights the importance of centring the patient, and their experience of their care event, during all healthcare episodes.”*
