# Supplementary material for: How value perspectives influence decision-making in the South African private healthcare sector: A cross-sectional comparative study
Source: PLoS One. 2025 Feb 4;20(2):e0316547. doi: 10.1371/journal.pone.0316547 (PMC11793816; doi:10.1371/journal.pone.0316547)
Supplement: Attachment — Submitted filename: Respone to reviews plos1 2024.12.10.docx [file pone.0316547.s004.docx]

**How value perspectives influence decision-making in the South African private**

**healthcare sector: a cross-sectional comparative study**

Response to reviewers

1) **The heading "introduction" on the abstract should be replaced with " Background"**

We have made this change.

2) **The sub-heading " importance of patient experience" in the introduction section of the paper is not necessary**

We have removed this sub-heading.

3) **Clarify the contribution of the study to related work in this area emphasizing advancement of knowledge on most recent work.**

We have added a section to the conclusion highlighting the unique contribution to the field made by this study. The section reads as follows:

*“While our findings support the centrality of clinical outcomes as the key determinant in the value equation, they highlight the importance of patient experience over event cost and make the argument for its inclusion into the tradition value equation. Our study further contributes to our knowledge of this subject by describing how: 1) relative value proportions of clinical outcome, patient experience and patient event cost when determining healthcare value; 2) clinical severity impacts value assignment; 3) value judgements differ between medical and surgical clinical scenarios, and 4) healthcare consumers and providers differ in assigning value – all within the South African healthcare context.”*

4) **Page 14" while it was negatively corrected" should be replaced with " while it was negatively correlated**

We have corrected this to read “while it was negatively correlated”.
